# Poor Adherence to the Screening-Based Strategy of Group B Streptococcus Despite Colonization of Pregnant Women in Greece

**DOI:** 10.3390/pathogens10040418

**Published:** 2021-04-01

**Authors:** Maria Maroudia Berikopoulou, Aikaterini Pana, Theodota Liakopoulou-Tsitsipi, Nikos F. Vlahos, Vasiliki Papaevangelou, Alexandra Soldatou

**Affiliations:** 1 School of Medicine, National and Kapodistrian University of Athens, 11527 Athens, Greece; 2 Department of Pediatrics, General Hospital of Sparta, 23100 Sparta, Greece; panakaterina@yahoo.gr; 3 Department of Pediatrics, Iaso Hospital, 15123 Athens, Greece; paidon.info@iaso.gr; 4 2nd Department of Obstetrics and Gynecology, School of Medicine, National and Kapodistrian University of Athens, Aretaieio Hospital, 11528 Athens, Greece; nikosvlahos@med.uoa.gr; 5 3rd Department of Pediatrics, School of Medicine, National and Kapodistrian University of Athens, Attikon University Hospital, 12462 Athens, Greece; vpapaev@med.uoa.gr; 6 2nd Department of Pediatrics, School of Medicine, National and Kapodistrian University of Athens, Children’s Hospital “Pan. & Aglaia Kyriakou”, 11527 Athens, Greece; alsoldat@med.uoa.gr

**Keywords:** GBS, maternal carrier status, adherence, culture, pregnancy, risk factors

## Abstract

Group B streptococcus (GBS) is a leading cause of serious neonatal infections. Maternal GBS colonization is associated with early- and late-onset neonatal disease (EOD/LOD). In Greece, a screening-based strategy is recommended, in which concurrent vaginal-rectal cultures should be obtained between 36 0/7 and 37 6/7 weeks’ gestation. We sought to examine the level of adherence to the GBS screening guidelines and estimate the prevalence of GBS colonization among pregnant women. Although in Greece the screening-based strategy is followed, we also examined known EOD risk factors and linked them to GBS colonization. A cross-sectional study of 604 women postpartum in three hospitals and maternity clinics was conducted. Following written informed consent, data were collected via a short self-completed questionnaire and review of patients’ records. In 34.6% of the enrolled pregnant women, no culture had been taken. Of the remaining, 12.8% had proper vaginal-rectal sample collections. The overall maternal colonization rate was 9.6%. At least one risk factor for EOD was identified in 12.6% of participants. The presence of risk factors was associated with positive cultures (*p* = 0.014). The rate of culture collection did not differ between women with or without an EOD risk factor. Adherence to a universal screening of pregnant women with vaginal-rectal cultures was poor. Despite probable underestimation of GBS carrier status, almost 1 in 10 participants were GBS positive during pregnancy. Screening of women with risk factors for EOD should, at least, be prioritized to achieve prevention and prompt intervention of EOD.

## 1. Introduction

Group B streptococcus (GBS) is one of the most common causes of postpartum and neonatal infections. GBS may colonize the gastrointestinal tract, especially the colon, whereas in pregnant women secondary colonization of the vagina and urethra occurs [1]. Neonates born to colonized mothers have a 50% chance of becoming carriers by ascending transmission or during their passage from the birth canal. Most carriers have no symptoms; only 2% are affected and present with early-onset disease (EOD) in the first week of life [2]. Although EOD may present in variable ways, sepsis accounts for almost 80% of the cases, pneumonia for 10%, and meningitis for 7% [3,4,5]. The incidence of EOD is estimated at 0.23 cases per 1000 pregnancies worldwide, which indicates that EOD is not that common. However, the mortality rate appears to be relatively high at 6.9% [6]. In Greece, a recent study revealed a lower incidence at approximately 0.17 cases per 1000 live births with a similar mortality rate [7]. According to the Center for Disease Control and Prevention (CDC) guidelines, the administration of intrapartum antimicrobial prophylaxis (IAP) to colonized pregnant women has been shown to reduce the EOD incidence in neonates [8,9,10].

The worldwide prevalence of maternal vaginal-rectal GBS colonization has risen to 18%. Maternal colonization rates differ regionally; the highest rates are observed in the Caribbean (35%) and the lowest in East Asia (11%) [11]. In Greece, the latest study of GBS burden was conducted in 2003 and estimated the colonization rate at 6.6%, which is much lower than the current global rates [12].

There are two approaches regarding the administration of IAP to pregnant women: The first is a selective risk-based strategy in which IAP is offered to women with known risk factors for EOD. These include preterm birth (<37 weeks’ gestation), preterm or prolonged rupture of membranes (>18 h), GBS bacteriuria during any trimester of the current pregnancy, intrapartum fever (>38 °C), and a history of a previous neonate with invasive GBS disease [5,13]. This strategy is currently in use in the Netherlands, the UK, and India, among other countries. The second approach is based on universal antepartum screening, in which vaginal-rectal cultures are obtained from all pregnant women between 36 0/7 and 36 6/7 weeks’ gestation; this specific timing for screening provides a 5-week frame, in which the culture results are considered valid, and includes births up to 41 gestation weeks. A swab from the lower vagina near the introitus and then from the rectum without the use of a speculum is obtained [5,7,8,13]. Therefore, in many countries, including the USA, Greece, as well as most other European countries, IAP is offered according to the screening results.

The rate of GBS colonization among pregnant women in Greece has not been studied for almost two decades, and the local level of adherence to universal GBS screening guidelines is unknown. Thus, the primary aim of this prospective cross-sectional study was to estimate the level of adherence to screening guidelines. Secondary aims of the study were: (a) to examine if the presence of EOD risk factors improved the rate of adherence to screening in the event of poor adherence to the recommended universal screening approach, and (b) to estimate the prevalence of GBS colonization among pregnant women with or without risk factors.

## 2. Results

A sample size of 604 participants was calculated for adequate power for this study. All 604 women invited to participate in the study were included.

### 2.1. Demographics

The mean age (±SD) of the participants was 34.2 ± 5.4 years. Of the 604 women, 508 (84.1%) were Greek, 47 (7.8%) were Albanian, and 49 women (8.1%) belonged to various ethnic groups. Most women lived in Attica (500 women; 84.4%) and were married (575 women; 95.2%). Over half of them (336 women; 55.6%) worked in the private sector, and 144 (23.8%) were unemployed. Finally, 70.5% of the participants (426 women) had public insurance, and 3.6% (22 women) were uninsured.

### 2.2. Interview Data

Approximately half of the participants (307 women; 50.8%) were primipara. Regardless of the place of delivery (private or public), most participants (520 women; 86.1%) were followed by an obstetrician privately during gestation. Most women (515 women; 85.9%) reported a once-monthly visit to their doctor.

Overall, 110 women (18.2%) reported health issues during the pregnancy, mainly gestational diabetes (47; 42.7%). None reported a history of giving birth to a neonate with invasive GBS disease. Finally, 139 women (23%) did not recall having had a GBS culture taken, while 102 women (16.9%) were unsure and did not answer the question. Of those who recalled having had a culture taken, 38 reported being positive for GBS.

### 2.3. Review of Medical Records

Over one in three participants (209 women; 34.6%) did not undergo GBS screening during pregnancy. GBS was isolated in 38 women, therefore, the colonization rate was estimated at 9.6% (95%; CI 6.7–12.5%). In 109 participants (27.9%), a GBS vaginal culture was recorded; 13 women (11.9%) were GBS carriers. Importantly, concurrent vaginal-rectal cultures were recorded in 50 women (12.8%); no woman in this group was GBS colonized. In 231 women (59.3%), the sample site was not recorded at all. However, of those 231 women, 121 recalled a vaginal sample during the interview.

Of the 604 women, 354 (58.6%) gave birth by cesarean section. Premature birth (<37 weeks’ gestation) occurred in 59 women (9.8%). In 14 cases (2.3%), prolonged rupture of membranes (>18 h) was recorded, and in one (0.2%), fever was documented during labor. Most participants (562 women; 93%) had negative urine cultures, and nine women (1.5%) had documented GBS bacteriuria during the current pregnancy. Collectively, 76 out of 604 (12.6%) had at least one risk factor for EOD.

### 2.4. Data Analysis

GBS culture results, according to interviews and medical records, did not differ. No woman with negative culture responded positively, and no colonized woman responded negatively.

There were no significant differences between GBS negative and colonized women in regard to age (*p* = 0.353), ethnicity (*p* = 0.829), marital (*p* = 0.583) and insurance status (*p* = 0.916), number of prenatal visits (*p* = 0.686), health issues during previous (*p* = 0.604), and current pregnancy (*p* = 0.844) or route of delivery (*p* = 0.482). The colonization rate was significantly higher among public employees (*p* < 0.001). Women with multiparity (>2 previous births) were more likely to be colonized (*p* = 0.008). A significant association was found between colonization and the presence of EOD risk factors; women with at least one risk factor were more likely to be colonized (*p* = 0.014). Among all risk factors examined individually, GBS bacteriuria during any trimester’s gestation was the only one associated with a positive culture outcome (*p* < 0.001) Detailed results are shown in Table 1. Finally, vaginal cultures were associated with the growth in GBS (*p* = 0.034).

Following stepwise a multiple logistic regression analysis, the presence of EOD risk factors and multiparity remained significantly associated with GBS colonization. Detailed results are shown in Table 2.

Participants delivering at public hospitals were more likely to have GBS cultures taken during pregnancy (*p* < 0.001).

Finally, the presence of EOD risk factors was not associated with the rate of GBS screening culture (*p* = 0.939). Of 76 women with at least one risk factor, 26 (34.2%) did not have a culture obtained.

## 3. Discussion

In this cohort study of 604 postpartum women, poor adherence to universal prepartum screening of GBS guidelines [5] was observed. Specifically, 34.6% (209 women) did not have a GBS culture taken at all, as their obstetricians did not suggest so. Among 395 women who were screened, almost 1 in 10 participants were GBS carriers. However, this finding possibly underestimates the true prevalence of GBS colonization since only 12.8% of women screened underwent concurrent vaginal-rectal sampling. Antepartum GBS screening in this population of women in Greece was inadequate, suggesting there is a lot of room for improvement. The adherence level observed in our study was approximately 65%. Compared to other European countries, this is mediocre with the highest rates recorded in France (89–96%) [14] and the lowest in Germany (22.7%). This low compliance may be justified by the fact that GBS screening in Germany is not covered by health insurance [15], a problem that does not occur in Greece. Three additional studies from the USA revealed a wide heterogeneity among states: high adherence levels were recorded in Tennessee (84.7%) and Minnesota (88.4%) [16,17], and mediocre in California (69.05%) [18]. Moreover, compared to our results, a higher rate of adherence was observed in Australia (76%) [19].

A possible explanation for this poor adherence could be the high proportion of cesarean sections in Greece. Indeed, one study carried out in Brazil justified the low GBS adherence levels due to the high proportion of cesarean sections [15]. However, according to the American College of Obstetricians and Gynecologists (ACOG) 2020 guidelines, women with a planned cesarean birth should also undergo an antepartum GBS culture, as a rupture of membranes could take place before planned birth [8].

The GBS colonization rate was estimated at 9.6% in this population of women. The latest investigation of GBS burden in Greece was conducted in 2003 and estimated the colonization rate at almost 7% by obtaining the concurrent vaginal-rectal swab [12]. Higher rates have been reported in other countries; the worldwide prevalence of maternal vaginal-rectal colonization with GBS was estimated at 18% with regional variations (11–35%) [6].

Another important finding of our study was that only 12.8% of the participants (50 women) reported culture vaginal-rectal specimens for GBS. In the remaining participants, a vaginal culture was reported causing the underestimation of GBS prevalence. Studies showed that vaginal-rectal sampling increased the detection of GBS carriers, compared to only vaginal sampling [20,21,22,23]. Surprisingly, in this study, women with vaginal cultures only were more likely to be found GBS carriers (*p* = 0.034). However, this is most probably a random finding and not a true superiority of vaginal sampling.

Based on the estimated GBS colonization rate (9.6%; 95%; CI 6.7–12.5%), the number of possible missed carriers was calculated. Out of the 209 women without a culture, 20 women (95%; CI 14–26.2%) could be colonized with GBS. This number could be even larger, as the rate of colonization (9.6%) observed may already be underestimated.

As already mentioned, a selective risk-based strategy, in which IAP is offered to women with known EOD risk factors, is recommended in other countries. This approach is based on the recognized association of these risk factors to being a GBS carrier [5,13]. In this study, we examined the EOD risk factors and the association to GBS colonization, as poor adherence to universal screening guidelines was suspected from the beginning, in an attempt to examine if the presence of EOD risk factors improved the rate of adherence to screening.

Indeed, in our study, women with at least one EOD risk factor were more likely to be colonized (*p* = 0.014); in fact, they were 2.75 times more likely to be carriers than those without a risk factor. Following individual examination of risk factors, the one with the strongest association with a positive screen was the GBS bacteriuria during any trimester’s gestation (*p* < 0.001). None of the other risk factors were associated individually. However, studies have shown a strong association between every EOD risk factor and maternal and neonatal GBS colonization [24,25]. Our findings could be explained due to the small sample size, the low prevalence of risk factors, and the low adherence levels with the universal screening guidelines. Another potential explanation of our findings is that GBS bacteriuria was an alarming finding for obstetricians, who responded with higher adherence to GBS screening than expected, even though screening in case of GBS bacteriuria was not necessary. Since GBS bacteriuria is considered as heavy colonization and is associated with an increased risk of developing pyelonephritis, anogenital tract colonization, and preterm labor [26], IAP is indicated in all cases [8].

Disappointingly, the rate of culture collection did not differ between women with or without an EOD risk factor. Specifically, almost 35% of the participants did not undergo GBS screening despite having at least one risk factor. This not only suggests poor adherence but also non-prioritization of higher-risk women.

Another significant finding was that the women delivering at public university maternity departments were more likely to undergo a GBS screening than those at the private hospital (*p* < 0.001). This difference may be attributed to the fact that obstetricians serving at teaching institutions might be more careful with adhering to guidelines and providing evidence-based care to pregnant women. However, this finding may not be generalizable to non-university-affiliated public obstetric departments in the country. Nonetheless, a systematic review comparing the general performance between public and private health care in the European Union strengthens our finding. Evidence from several countries, including Greece, proved that public hospitals are as efficient as, or sometimes even more efficient than private ones [27].

There are several limitations of our study. First, the GBS colonization rate calculated may be underestimated for the following reasons: (a) a significant number of participants did not undergo screening; (b) most screening samples were only vaginal; (c) the timing of the cultures obtained, and culture conditions were not found in the medical records reviewed. Indeed, omission of the vaginal-rectal sampling, the timing of cultures more than five weeks prior to delivery, and lack of culture enrichment with selective broths may lead to increased false-negative results [7,21,22,23,28]. However, an increased rate in GBS colonization in the Greek population of women compared to the previously known rate was highlighted. In addition, this study clearly indicated poor adherence to current universal screening guidelines in Greece and inadequate consideration of maternal risk factors for EOD.

## 4. Materials and Methods

### 4.1. Study Design

A multicenter hospital-based cohort study was conducted at three major hospitals in Athens, specifically two public university-obstetric departments and a private maternity hospital from 1 November 2019 to 30 June 2020. The study protocol was approved by the Hospital Ethics Committees of all three hospitals. Written informed consent was obtained from all eligible participants prior to study enrolment.

### 4.2. Study Population

Based on the previous colonization rate of GBS in pregnant women in Greece (6.6%) and the desired study parameters (accuracy: 2%, *p*-value: 5%, power: 80%), a sample size of 604 participants was calculated (339 from private and 265 from public hospitals). The proportion of participants from private and public hospitals was determined according to the statistics provided by the Hellenic Statistical Authority regarding the number of births in the private and public sectors in Greece during 2018.

### 4.3. Measurements

Clinical data were collected on three working days a week. Initially, participants were interviewed based on a structured questionnaire. Information obtained included demographic data, marital and insurance status, the number of prenatal visits, previous obstetric history, the results of urine, and GBS screening cultures if known. Additional data were obtained from the patients’ medical records concerning obstetric history, the presence of EOD risk factors, and GBS status (site of sampling and cultures’ results).

### 4.4. Statistics

Quantitative variables were calculated in mean values and standard deviations (SD). Qualitative variables were expressed in absolute (N) and relative (%) frequencies. Pearson’s x2 test or Fisher’s exact test were used to comparing ratios where necessary. The student’s t-test was used to compare quantitative variables between two groups.

A stepwise multiple logistic regression analysis (*p* for removal was set at 0.1 and p for entry was set at 0.05) was conducted in order to investigate independently associated factors with the existence of positive GBS. Adjusted odds ratios with 95% confidence intervals were computed from the results of the logistic regression analysis. All *p*-values reported are two-tailed.

Statistical significance was set at 0.05. Statistical analysis was conducted using SPSS statistical software (version 23.0).

## 5. Conclusions

In conclusion, in a sizable population of postpartum women, an increased rate of GBS colonization compared to the past was found, as well as inadequate adherence to universal GBS screening guidelines. Although current evidence-based guidelines for universal maternal GBS screening should be followed, at least screening among high-risk women should be prioritized; only then would EOD be prevented, and prompt intervention secured. Therefore, an urgent need for targeted interventions is necessary to improve the quality of care provided to expectant mothers.

## Figures and Tables

**Table 1:** Associations between early-onset disease (EOD) risk factors and culture result.

EOD Risk Factors		GBS Positive (%)	GBS Negative (%)	*p*-Value
Preterm birth	No	10.5	89.5	0.066
	Yes	0	100	
Preterm or prolonged rupture of membranes	No	9.7	90.3	0.978
	Yes	10	90	
GBS bacteriuria	No	8.2	91.8	<0.001
	Yes	77.8	22.2	
Intrapartum fever	N.A.	0	0	N.A.
History of a previous neonate with invasive GBS disease	N.A.	0	0	N.A.
At least one EOD risk factor	No	6.8	93.2	0.014
	Yes	17.2	82.8	

**Table 2:** Independently associated factors with a positive GBS culture following stepwise multiple logistic regression analysis.

	GBS Negative	GBS Positive	*p*-Value	Odds Ratio (95% CI)
**Multiparity** (>2 previous births)	76	24	0.008	0.33 (0.14–0.78)
**At least one EOD risk factor**	82.8	17.2	0.014	2.75 (1.20–6.30)

## Data Availability

The data presented in this study are available on request from the corresponding author. The data are not publicly available due to anonymity and confidentiality.
